# Effects of 5-Methyl-2′-Deoxycytidine in G-Quadruplex Forming Aptamers d(G_3_C)_4_ and d[GCG_2_(CG_3_)_3_C]: Investigating the Key Role of the Loops

**DOI:** 10.3390/biom15060753

**Published:** 2025-05-23

**Authors:** Veronica Esposito, Daniela Benigno, Carla Aliberti, Camilla Esposito, Elisabetta Panza, Antonella Virgilio, Aldo Galeone

**Affiliations:** Department of Pharmacy, University of Naples Federico II, Via D. Montesano 49, I-80131 Naples, Italy; verespos@unina.it (V.E.); daniela.benigno@unina.it (D.B.); carla.aliberti@unina.it (C.A.); camilla.esposito3@unina.it (C.E.); e.panza@unina.it (E.P.); galeone@unina.it (A.G.)

**Keywords:** aptamers, G-quadruplex, 5-methyl-2′-deoxycytidine, antiproliferative activity

## Abstract

T40214 (STAT) and its recently investigated analogue STATB are G-quadruplex (G4) forming aptamers characterized by an unusually high percentage of C. The therapeutic potential of T40214 relies on its ability to inhibit the signalling pathway of STAT3, a protein frequently overexpressed in tumor cells. STAT adopts a dimeric 5′-5′ end-stacked quadruplex structure, characterized by parallel strands, three G-tetrads and three propeller-shaped loops formed by a cytidine residue. STATB folds in a very similar structure, apart from an additional cytidine bulge loop. Many studies suggest that thermal stability and topology of G4 can be significantly affected by C methylation, thus resulting in altered interaction of G4-binding proteins with these structures. Considering this, two series of STAT and STATB analogues containing a single 5-methyl-2′-deoxycytidine (**mC**) residue instead of canonical C nucleotide in the loop have been prepared and investigated by a combination of spectroscopic and electrophoretic techniques. CD, NMR and PAGE data clearly indicate that all derivatives adopt dimeric G4 strictly similar to that assumed by parent aptamers, but with higher stabilities. Furthermore, the resistance to nucleases and the antiproliferative activity of these **mC**-containing derivatives against HCT116 (human colorectal carcinoma) and T24 (human bladder carcinoma) cell lines have been evaluated. In most of the cases, STAT and STATB derivatives inhibit cell proliferation to different extents, although to a lesser degree than the unmodified parent sequences. All the data highlight the key role of the loops and indicate **mC** as a useful tool to contribute favorably to the stability of G4-forming aptamers without alteration of their topology, required for the biological activity.

## 1. Introduction

Aptamers are small DNA- or RNA-based oligonucleotides, generally identified by several miscellaneous techniques jointly called SELEX (systematic evolution of ligands by exponential enrichment) [1]. Thanks to their peculiar properties, aptamers are able to bind a given target (a small molecule as well as a protein or other molecules) with high affinity and selectivity. Under suitable conditions, aptamers adopt distinctive three-dimensional structures whose thermal stability is of crucial importance for their therapeutic or diagnostic development and application. For these reasons, a non-negligible number of aptamers show G-rich sequences that, in turn, promote the folding in G-quadruplex structures (G4s), being among the most stable secondary conformations adopted by nucleic acid sequences [2,3]. G4s are stabilized by the stacking interactions of structural units called G-tetrads, in which four guanines are assembled in a planar arrangement by Hoogsteen hydrogen bonding. The nature of G4s is broadly polymorphic, since they usually can originate from one, two, or four separate strands of DNA or RNA arranged in various orientations such as parallel, antiparallel, or hybrid. Furthermore, they differ in loop size and sequence that can affect the structural and physical–chemical properties.

Among the G4-adopting DNA aptamers, the most occurring nucleosides in the loop regions are thymidine and deoxyadenosine, while deoxyguanosine and deoxycytidine are by far less present [3]. This piece of data is not particularly surprising, considering that deoxyguanosines in the loop regions could become part of the G-runs, thus increasing G4 polymorphism. On the other hand, the presence of deoxycytidines in the G4-aptamer sequence could cause the formation of canonical C-G base pairs, thus favoring the formation of double-stranded structures to the disadvantage of the G4 ones. For this reason, the number of deoxycytidines in most of the sequences of known G4-adopting aptamers does not exceed 20% [3]. However, sometimes higher percentages of deoxycytidines can be observed in the case of naturally occurring G4s. For example, the sequence WT22 (G_3_C_2_AC_2_G_3_CAG_5_CG_3_), derived from the WNT1 promoter region, shows a content of deoxycytidines of about 27% [4]. As is well known, deoxycytidine methylation constitutes an epigenetic mark that plays a crucial role in the modulation of transcriptional activity and other genome processes. Considering the well-ascertained involvement of G4s in gene expression and regulation, several studies have been devoted to investigating the effects of cytosine methylation on the properties of biologically relevant G4s [5,6], such as the G4s associated with the fragile X syndrome d(CGG)_n_ nucleotide repeats [7,8] and the C9orf72 repeat [9]. Further similar investigations have concerned the impact of the 5-methyl-2′-deoxycytidine (**mC**) in G4-forming sequences related to the *bcl-2* promoter [10] and the first exon of the human telomerase reverse transcriptase gene (*hTERT*) [11]. Analogously, more recent studies have involved other meaningful G4s, such as those derived from the promoter regions of two cancer-related genes, namely *WNT* [12] and *c-kit* [13] and the dopamine transporter gene (*DAT1*) [14]. The whole of the data emerging from the above investigations and other similar studies [15] suggest that thermal stability and topology properties of G4s can be significantly affected by C methylation, thus resulting in altered interaction of G4-binding proteins with G4s.

**Figure 1:**
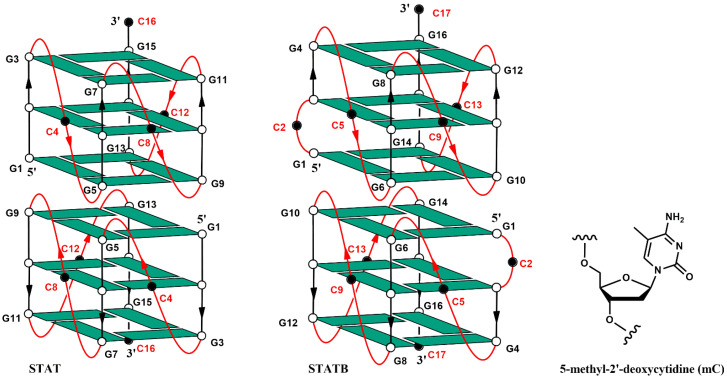
Schematic representation of the dimeric parallel-stranded G-quadruplexes formed by STAT and STATB. White circles and green parallelograms indicate anti 2′-deoxyguanosines. Black circles in the loop regions (in red) indicate deoxycytidines.

Among the G4-forming aptamers, T40214 (STAT) [16,17] and its recently investigated analogue STATB [18] represent some remarkable deviations from the trend generally observed related to the low contents of cytidine, since their sequences (G_3_CG_3_CG_3_CG_3_C and GCG_2_CG_3_CG_3_CG_3_C, respectively) contain a higher percentage of C than usual (25% and 29%, respectively). The therapeutic potential of T40214 relies on its ability to inhibit the signaling pathway of STAT3, considering that this protein is frequently overexpressed in tumor cells as well as tissue samples, and regulates the expression of numerous oncogenic genes controlling the growth and metastasis of tumor cells [19]. Aptamer STAT adopts a dimeric 5′-5′ end-stacked structure, in which each G4 shows a topology characterized by parallel strands, three G-tetrads and three propeller-shaped loops, each of whom is formed by a cytidine residue [20]. Aptamer STATB folds in a very similar structure, apart from an additional bulge loop formed by the extra cytidine in the second position of the sequence (Figure 1) [18].

Taking into account the ability of **mC** to affect both the topological preference and the interaction with the proteins of several biologically relevant G4s, it would also be worth investigating the effects of the presence of **mC** in therapeutically promising G4-aptamers. In this frame, two series of STAT and STATB analogues containing a single **mC** residue instead of the canonical C nucleotide in the loop regions have been prepared and investigated in terms of their structural properties by a combination of spectroscopic and electrophoretic techniques (Table 1). Furthermore, the resistance to nucleases and the antiproliferative activity of these **mC**-containing derivatives against HCT116 (human colorectal carcinoma) and T24 (human bladder carcinoma) cell lines have been evaluated.

## 2. Materials and Methods

### 2.1. Oligonucleotide Synthesis and Purification

The oligonucleotides reported in Table 1 were synthesized on a K&A H-16 DNA synthesizer (K&A Labs GmBH, Schaafheim, Germany) using solid phase β-cyanoethyl phosphoramidite chemistry at 5 µmol scale. A modified monomer was incorporated in the sequences using commercially available N4-acetyl-5-methyl-5′-O-(4,4′-dimethoxytrityl)-2′-deoxycytidine-3′-cyanoethyl phosphoramidite (Merck KGaA, Darmstadt, Germany). For STAT M5 and STATB M5, a universal support was employed. The oligomers were cleaved from the support and deprotected using concentrated aqueous ammonia at 55 °C overnight. The resulting mixtures were concentrated under reduced pressure, redissolved in water, and subsequently analyzed and purified via high-performance liquid chromatography (HPLC) on a Nucleogel SAX column (Macherey-Nagel, Düren, Germany, 1000-8/46), using buffer A: 20 mM NaH_2_PO_4_/Na_2_HPO_4_ aqueous solution (pH 7.0) containing 20% (*v*/*v*) CH_3_CN and buffer B: 1 M NaCl, 20 mM NaH_2_PO_4_/Na_2_HPO_4_ aqueous solution (pH 7.0) containing 20% (*v*/*v*) CH_3_CN, applying a linear gradient from 0 to 100% B for 45 min at a flow rate of 1 mL/min. Collected fractions were desalted using C-18 Sep-Pak cartridges and the purity of the isolated oligonucleotides was confirmed to exceed 98% by NMR spectroscopy.

### 2.2. CD Spectroscopy

CD samples of oligonucleotides reported in Table 1 were prepared at ODN final concentration of 25 µM in 10 mM potassium phosphate buffer (KH_2_PO_4_/K_2_HPO_4_) with 70 mM KCl, pH 7.0 followed by annealing heating at 90 °C and slow cooling to room temperature. CD spectra of all quadruplexes and CD melting curves were acquired using a Jasco 715 CD spectrophotometer (Jasco, Tokyo, Japan). CD spectra were recorded from 320 to 220 nm at 100 nm min^−1^ scan rate, with a response of 4 s, at 1.0 nm bandwidth. All spectra were baseline-corrected by subtracting the buffer signal. The temperature was kept constant at 20 °C with a thermoelectrically controlled cell holder (Jasco PTC-348). CD melting curves were obtained by monitoring the CD signal as a function of temperature (range: 20–95 °C) for all G-quadruplexes, annealed as previously reported, at their maximum Cotton effect wavelengths. To test the G-quadruplex thermal stabilities at low potassium concentration, samples of all ODNs were prepared at an ODN concentration of 25 µM, using a potassium phosphate buffer 1 mM KH_2_PO_4_/K_2_HPO_4_, 5 mM KCl, pH 7.0, and annealed as above. The CD data were recorded using a scan rate of 30 °C/h in a 0.1 cm pathlength cuvette.

### 2.3. NMR Spectroscopy

NMR samples were prepared at a concentration of ~1 mM in 0.6 mL (H_2_O/D_2_O 9:1 *v*/*v*) of buffer solution with 10 mM KH_2_PO_4_/K_2_HPO_4_, 70 mM KCl, and 0.2 mM EDTA (pH 7.0). Samples were heated for 5–10 min at 90 °C and slowly cooled to room temperature over 10–12 h, followed by equilibration at 4 °C for several hours. Completion of the annealing was confirmed by superimposability of the ^1^H NMR spectra over time. NMR spectra were acquired at 25 °C using a 700 MHz Bruker spectrometer (Bruker-Biospin, Billerica, MA, USA). Proton chemical shifts were referenced to the residual water signal, resonating at 4.78 ppm (25 °C, pH 7.0). Water suppression was achieved using excitation sculpting with the gradient routine included in the “zgesgp” pulse sequence [21]. NMR data processing was performed by using the vendor software TOPSPIN 4.1.4 (Bruker Biospin Gmbh, Rheinstetten, Germany).

### 2.4. Gel Electrophoresis

All ODNs were analyzed by non-denaturing polyacrylamide gel electrophoresis. Samples were prepared at an ODN concentration of 1 mM by using a potassium phosphate buffer 10 mM KH_2_PO_4_/K_2_HPO_4_, 70 mM KCl, pH 7.0, and submitted to the annealing procedure (heating at 90 °C and slowly cooling at room temperature). Each sample was loaded on a 20% polyacrylamide gel containing Tris–Borate-EDTA (TBE) 2.5× and KCl 20 mM. The electrophoresis was performed using 1× TBE running buffer supplemented with 50 mM KCl. A small volume of glycerol/10× TBE was added to each sample before loading. Electrophoresis was conducted at 8 V/cm at approximately 10 °C and bands were visualized using UV shadowing.

### 2.5. Isothermal Association Kinetics

The kinetics of association of unfolded oligonucleotides into well-defined G4 structures was examined for each sequence in a potassium phosphate buffer (1 mM, pH 7.0), including 5 mM KCl. The experiments were acquired at 25 µM ODN concentration. Each sample solution was kept at 95 °C for 5 min to allow for the dissociation of any partially folded species present. The structural transition from the unfolded species to the G4 folded ones was monitored at constant temperature (20 °C) by recording the increase in CD signal intensity for all G4s at their maximum Cotton effect wavelengths as a function of time.

### 2.6. Nuclease Stability Assay

The stability of the oligonucleotides in biological conditions was evaluated in 10% Fetal Bovine Serum (FBS) diluted with Dulbecco’s Modified Eagle’s Medium (DMEM) at 37 °C and studied by CD analysis. Approximately 7 nmol of stock solution of each ODN (~1 O.D.U.) was dried under reduced pressure and then incubated with 250 μL 10% FBS at 37 °C. CD signal reduction was monitored over time to assess degradation. The CD amplitude at 264 nm was plotted against the reaction time. CD spectra at 0, 24, 48 and 72 h for all ODNs were recorded at 37 °C using a Jasco 715 spectrophotometer equipped with a Peltier temperature control system (Jasco, Tokyo, Japan). CD spectra were acquired from 320 to 240 nm with a 1 s response time and a 1 nm bandwidth using a 0.1 cm quartz cuvette. Data were corrected by subtracting the signal of the reaction medium (10% FBS in DMEM).

### 2.7. MTT Assay

Human colon cancer cell line HCT116 (cat. no. CCL-247) and human bladder cancer cell line T24 (cat. no. HTB-4) were acquired from the American Type Culture Collection (ATCC, Manassas, VA, USA). Cells were cultured in DMEM (Sigma-Aldrich, Milan, Italy; cat. no. D6546) supplemented with 10% fetal bovine serum (FBS) (Gibco, Milan, Italy; cat. no. A4736301), penicillin (100 U/mL), and streptomycin (100 μg/mL) (cat. no. 30-002-CI), 2 mmol/L L-glutamine (cat. no. 25-005-CI), and 0.01 M HEPES buffer (cat. no. 25-060-CI) (all from Corning, Manassas, VA, USA), and placed at 37 °C in a humidified incubator containing 5% CO_2_. Cells were seeded into 96-well plates (3 × 10^3^ cells/well) and adhered overnight. Cells were treated with STAT, STATB and their analogues (10 and 30 µM) for 72 h. A volume of 100 μL/well of MTT (3-(4,5-dimethylthiazol-2-yl)-2,5-diphenyltetrazolium bromide, cat. M5655, Merck, Italy) (final concentration 0.25 mg/mL in DMEM) was added and incubated for 3 h at 37 °C. After this time, MTT was removed, and the formed purple formazan crystals were dissolved in 100 μL/well DMSO. The absorbance was measured at 540 nm by a microplate spectrophotometer reader (Thermo Scientific Multiskan GO, Thermo Fisher Scientific, Waltham, MA, USA).

## 3. Results

### 3.1. CD Spectroscopy

Circular dichroism (CD) spectroscopy is a key tool for the characterization of G4s. Different G-quartet stacking, strand orientation and loop arrangements generate diverse G4 topologies [22], each of which displays typical CD spectral profiles [23,24]. In general, excluding some exceptions, G4 CD spectra are characterized by λ_max_ ≈ 260 nm and λ_min_ ≈ 240 nm for parallel type, λ_max_ ≈ 290 nm and λ_min_ ≈ 260 nm for antiparallel type, and λ_max_ ≈ 295 nm and ≈260 nm, λ_min_ ≈ 245 nm for “hybrid” (or 3 + 1) type [25,26]. Therefore, CD spectrum analysis allows us to obtain preliminary structural information for G4s in solution.

**Figure 2:**
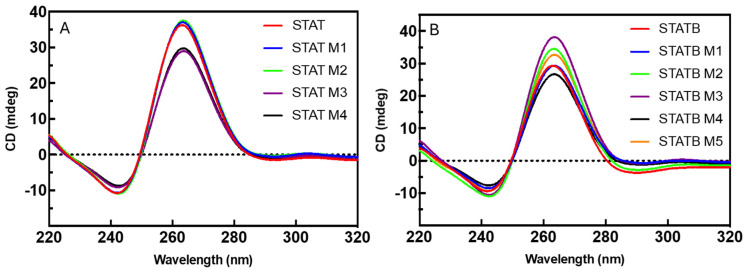
CD spectra at 20 °C of STAT (**A**) and STATB (**B**) analogues in potassium phosphate buffer 1 (10 mM KH_2_PO_4_/K_2_HPO_4_, 70 mM KCl, pH 7.0). See the Materials and Methods section for details.

To evaluate the folding topology of studied derivatives in comparison with those of their respective reference sequences (STAT and STATB), CD spectra were measured for each sequence, after appropriate samples annealing in two different potassium phosphate buffers (1: 10 mM KH_2_PO_4_, 70 mM KCl, pH 7.0; 2: 1 mM KH_2_PO_4_, 5 mM KCl, pH 7.0; Figure 2A,B and Figure S1A,B, respectively). All studied sequences in both buffer conditions showed the CD spectrum representative of a parallel G4 with all guanosines in *anti*-glycosidic conformation, that is, a CD profile with a negative band at 242 nm and a positive one at 264 nm. Furthermore, in potassium buffer 1 at the same ODN concentration, STAT M1 and STAT M2 on one side (Figure 2A) and STATB M1 on the other (Figure 2B) showed CD profiles perfectly superimposable to that of the parent ones, while for all the other derivatives, slight differences were observed in terms of CD signal intensity. These data clearly imply that the studied analogues fold in G4s strictly close to that of the aptamer T40214.

A further application of CD spectroscopy is the determination of the thermal stability of the G4 structures [27]. For this purpose, CD spectra were acquired by following the wavelength of maximum ellipticity for each sample over a temperature range of 20–95 °C.

CD melting experiments were initially acquired in potassium buffer 1 (Figure S2A,B), since it was the same used in biological assays. In these experimental conditions, for STAT and its derivatives (Figure S2A) it was not possible to determine sigmoidal heating profiles from which to deduce the melting temperatures (T_m_), since the intensity of the CD signal remains almost unchanged at least up to 80 °C. STATB and its analogues, on the other hand, showed well-defined melting profiles, all revealing T_m_ above 80 °C (Figure S2B).

To evaluate in depth the effect of the single dC methylation on the thermal stability of the G4s studied, CD melting curves were recorded for each sample annealed in buffer 2, i.e., at low potassium ion concentrations (Figure S3A,B). In these conditions, all the analyzed structures showed sigmoidal profiles, from which the T_m_s reported in Table 1 were obtained. It is interesting to note that all modified derivatives revealed T_m_s very similar to each other within the same series (≈89 °C for STAT series and ≈67 °C for STATB series), but higher than that of the respective parent G4s; therefore, the single substitution of a C residue in the STAT and STATB sequences with an **mC** residue not only preserves the structural folding of the original complexes, but also significantly contributes to stabilizing these structures in all sequence positions.

### 3.2. Nuclear Magnetic Resonance

**Figure 3:**
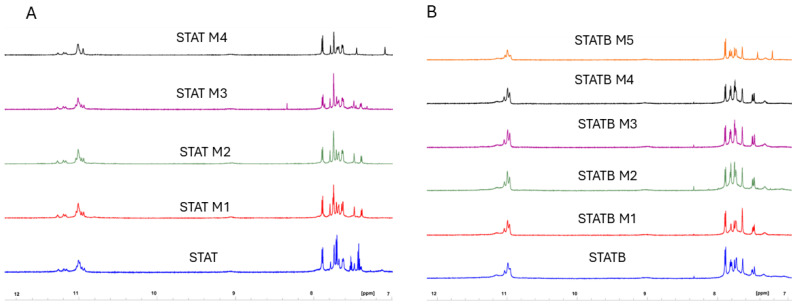
Imino and aromatic proton regions of the 1H-NMR spectra (700 MHz) of STAT (**A**) and STATB (**B**) series. See the Materials and Methods section for details.

The capability of all the derivatives here reported to adopt parallel dimeric G-quadruplexes like those of reference sequences was also assessed by NMR spectrometry. The 1H NMR spectra of each modified ODN containing a single **mC** replacing a C loop residue, one at a time, were separately examined after an appropriate annealing procedure in K^+^ containing buffer 1 and compared with the corresponding counterpart, STAT or STATB, endowed with all-cytidine loops (Figure 3A,B). As for STAT and STATB, G-quadruplex diagnostic imino signals were observed in the region between 10.5 and 12.0 ppm of the spectra (Figure S4), effectively underlining that all variants fold into dimeric quadruplex structures almost identical to those of their starting sequences, exhibiting superimposable imino proton profiles regardless of **mC** loop residue position in the quadruplex.

### 3.3. Polyacrylamide Gel Electrophoresis (PAGE)

To verify unequivocally the ability of the modified sequences to fold in dimeric structures as the original ones, we analyzed them using non-denaturing Polyacrylamide Gel Electrophoresis (PAGE) (Figure S5A,B), using STAT and STATB as references for each series, since they have already been proven to fold into very similar dimeric 5′-5′ end-stacked G4 structures [18]. As monomeric references, TTTT-STAT and TT-STATB were employed, since the introduction of extra thymidines in 5′ is a validated strategy to avoid the dimer formation [28]. The PAGE band migration pattern clearly indicated that all modified derivatives adopt dimeric G4 structures, since all of them move more slowly than the monomeric G4s used as references, while they co-migrate with STAT and STATB bands. These data confirmed for all studied analogues the aptitude to adopt dimeric G4s strictly similar to that assumed by the parent aptamers.

### 3.4. Association Kinetics

The kinetics of formation of the studied G4 complexes were estimated by monitoring their CD ellipticity at the maximum Cotton effect wavelengths as a function of time. Each sample was denatured by heating it at 95 °C for 5 min and rapidly cooling it at 20 °C. Kinetic profiles reported in Figure S6 reveal the increase in CD signal intensity over time due to the ongoing formation of structured species in solution. All STATB derivatives showed profiles closely similar to each other and almost superimposable to that of the parent one, thus indicating very high rates of formation. Indeed, after only 120 s in all cases, the formation process of G4 complex was completed. These data clearly indicate that the single substitution of a C residue in STATB sequence with an **mC** residue has no influence on the folding kinetics of the original complex. In the case of STAT and its derivatives, it was not possible to record association kinetics due to the high thermal stabilities of their G4 complexes (Tm above 80 °C), which prevents their complete initial denaturation under the experimental conditions used.

### 3.5. Nuclease Stability Assay

**Figure 4:**
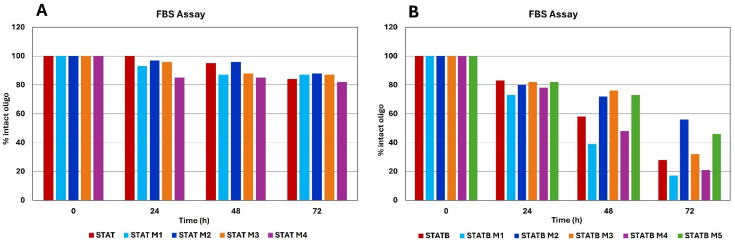
G-quadruplex stability of STAT (**A**) and STATB (**B**) analogues in 10% Fetal Bovine Serum (FBS) diluted with Dulbecco’s Modified Eagle’s Medium (DMEM), registered at different time at 37 °C. See the main text and the Materials and Methods section for details.

Despite their high biocompatibility, the therapeutic application of aptamers is limited by their susceptibility to hydrolysis by nucleases; however, chemical modifications can generally contribute to defending them from nuclease digestion [29]. Therefore, to evaluate their resistance in biological environments, all studied ODNs were tested in a degradation assay in fetal bovine serum (FBS) and examined by circular dichroism analysis [30,31] (Figure 4A,B, Figures S7 and S8). Since a hydrolysis of phosphodiester linkages implies a disruption of G-quadruplex structure, immediately evident in the CD spectra of the G4 analogues analyzed, CD spectra of all ODNs were recorded from 320 to 240 nm at 0, 24, 48 and 72 h at 37 °C in 10% FBS, subtracting the background scan (10% FBS in DMEM) (Figures S7 and S8). Each sample at different times retained the typical CD profile of parallel G4 in which all guanosines adopt anti-glycosidic conformations; therefore, the stability of G4s was monitored by the change in the CD signal intensity around 264 nm over time (Figure 4). A time-dependent reduction in undegraded species was observed for all complexes; however, STAT analogues, similarly to their natural counterpart, revealed a noteworthy stability to serum nucleases, since, in all cases, more than 80% of intact oligo remains up to 72 h (Figure 4A and Table S1). Instead, STATB derivatives showed a greater variability in terms of resistance in a biological environment than their parent one (Figure 4B and Table S1). Under the same experimental conditions, STATB M1 and STATB M4 revealed a lower nuclease resistance than STATB, since approximately 20% of their G4 structured species were still present at 72 h; STATB M3 is slightly more resistant to nucleases than the natural one, while a significant contribution of the single site-specific substitution was evident in the case of STATB M2 and STATB M5, given that they proved the persistence of about 55% and 45% of folded structures at 72 h, respectively.

These data revealed a significant biostability of all STAT analogues, strictly comparable to that of the natural one. Instead, in the case of STATB series, it is noteworthy the increased stability to serum nucleases of two derivatives, STATB M2 and STATB M5, compared to the unmodified sequence.

### 3.6. Antiproliferative Activity

**Figure 5:**
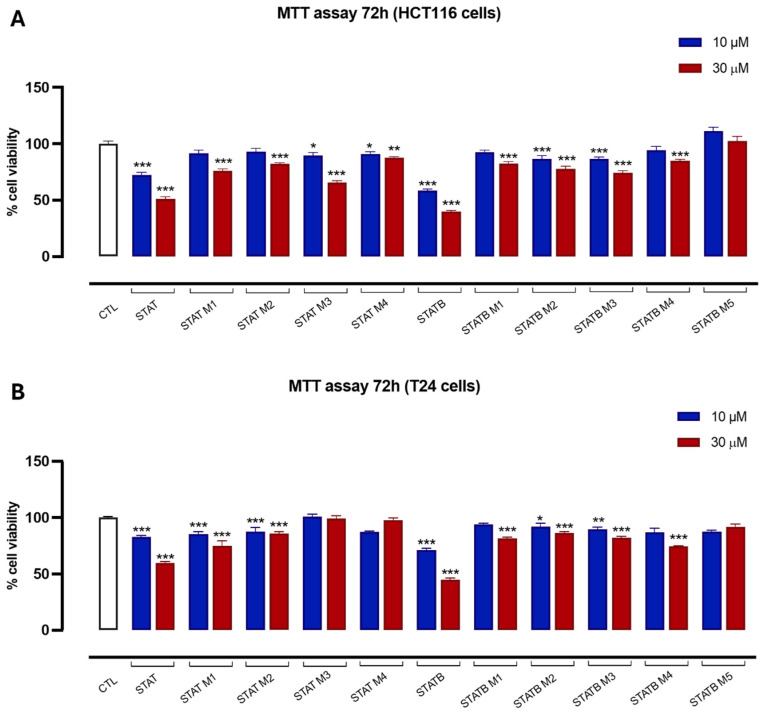
Effect of STAT and STATB series on HCT116 (**A**) and T24 (**B**) cell proliferation. Cell proliferation was measured using the MTT assay and evaluated at 72 h. Each experiment (*n* = 3) was run in quadruplicate. * *p* < 0.05; ** *p* < 0.01; *** *p* < 0.001 vs. CTL.

The potential anticancer activity of STAT, STATB, and their analogues was evaluated based on the proliferative capacity of HCT116 and T24 cells, which are well established in vitro models of human colon and bladder cancer, using the MTT assay. Both cell lines were treated with STAT and its four distinct derivatives, as well as STATB and its five analogues. Each compound was tested at concentrations of 10 and 30 µM for 72 h. Untreated cells served as the control. After 72 h, most compounds from the STAT and STATB series at 30 µM significantly inhibited cell proliferation, with inhibition percentages ranging from approximately 10% to 50% (Figure 5A,B). However, STATB M5 in both HCT116 and T24 cells, as well as STAT M3 and STAT M4 in T24 cells, showed no antiproliferative effect.

## 4. Discussion

In this study, we designed and prepared two series of G4 aptamers, STAT and STATB analogues, containing a single **mC** residue instead of the canonical C nucleotide in the sequences, and studied their structural and biological features, with the aim to evaluate the ability of **mC** to affect the topology of these G4s or their antiproliferative potential. Although it is known that loops, besides steric effects, are often involved in additional interactions that strongly influence the G-quadruplex folding [32], in our study CD, NMR and PAGE data clearly indicated that all derivatives adopt dimeric G4s strictly similar to that assumed by the parent aptamers, thus indicating that a single site substitution of C loop residue with **mC** does not alter the STAT and STATB general folding topology. However, under the given solution conditions, all analogues showed T_m_s higher than that of the respective parent G4s, thus confirming the important role of the loops in contributing significantly to the G-quadruplex stability [33].

Regarding the resistance in biological environments, data suggest that the presence of an **mC** residue in the loop can affect the biostability of the aptamers. Although, in the STAT series, no notable increase in biostability was observed compared to the natural sequence (probably because of its intrinsic high resistance to nucleases), in the STATB series, the favorable contribution of **mC** to the biostability of this G4 is evident when the single substitution involves specific positions, probably more susceptible to nuclease digestion, i.e., the bulge loops and the 3′-ends. In particular, the most interesting results concern the derivatives STATB M2 and STATB M5, for which approximately 50% of their structures remain unaffected for up to 72 h in FBS, thus proving a higher biostability compared to the unmodified aptamer showing a minor amount of residual intact structure (25–30%).

Furthermore, the antiproliferative activities of STAT, STATB, and their analogues were evaluated against HCT116 and T24 cancer cell lines. Except in the cases of STATB M5, STAT M3 and STAT M4, all tested compounds from both the STAT and STATB series inhibited cell proliferation at 30 µM to different extents, although, in both cell lines, the most active compounds of each series remained the unmodified parent sequences. These data clearly suggest a strong involvement of the loops and the 3′-ends in the interactions with the targets implicated in the antiproliferative activity of STAT and STATB, since the presence of a single methyl group, notwithstanding the retained original G4-folding topologies, significantly influences the cytotoxic activities of these aptamers. The importance of the loops for the biological activities observed in our study is in agreement with previous investigations involving other structurally different G4-aptamers endowed with antiproliferative activity as TBA [34,35] and AS1411 [36,37].

## 5. Conclusions

In short, we have observed that all STAT and STATB analogues studied preserve the dimeric G4s adopted by the parent aptamers; therefore, a single loop residue substitution with **mC** does not modify the STAT and STATB topology. Furthermore, all derivatives have a thermal stability higher than that of the parent ones, thus indicating the involvement of the loops in G-quadruplex stability. Cytotoxic data also suggest a key role of the loops in the antiproliferative activity of STAT and STATB, since all analogues containing only a single methyl group more than their natural counterparts are found to be less biologically active, while maintaining the same G4-topologies.

The whole of the data indicate **mC** as a useful tool to contribute favorably to the thermal and biological stability of G4-forming aptamers without alteration of their topology, being required for the biological activity. The reported data confirm the ability of **mC** to influence the interaction with the proteins not only in biologically relevant G4s, but also in therapeutically interesting sequences. Therefore, these results may be useful in the design and development of novel G4 aptamers as potential anticancer drugs.

## Figures and Tables

**Table 1:** Sequences and melting temperatures (T_m_) in low-concentration potassium buffer of the investigated ODNs (1 mM KH_2_PO_4_, 5 mM KCl, pH 7). **mC**: 5-methyl-2′-deoxycytidine. ∆T_m_ compared to the natural sequences STAT and STATB.

NAME	SEQUENCE (5′-3′)	T_m_ (±1)	∆T_m_
STAT	GGGCGGGCGGGCGGGC	82	-
STAT M1	GGG**mC**GGGCGGGCGGGC	90	+8
STAT M2	GGGCGGG**mC**GGGCGGGC	90	+8
STAT M3	GGGCGGGCGGG**mC**GGGC	87	+5
STAT M4	GGGCGGGCGGGCGGG**mC**	89	+7
STATB	GCGGCGGGCGGGCGGGC	63	-
STATB M1	G**mC**GGCGGGCGGGCGGGC	68	+5
STATB M2	GCGG**mC**GGGCGGGCGGGC	68	+5
STATB M3	GCGGCGGG**mC**GGGCGGGC	68	+5
STATB M4	GCGGCGGGCGGG**mC**GGGC	67	+4
STATB M5	GCGGCGGGCGGGCGGG**mC**	66	+3

## Data Availability

All data generated or analyzed during this study are included in this published article and its Supplementary Information Files.

## Supplementary Materials

The following supporting information can be downloaded at: https://www.mdpi.com/article/10.3390/biom15060753/s1, Figure S1: CD spectra at 20°C of STAT (A) and STATB (B) analogues in potassium phosphate buffer 2; Figures S2 and S3: CD melting profiles of analyzed ODNs in different buffer conditions; Figure S4: Imino proton regions of the 1H-NMR spectra (700 MHz) of STAT (A) and STATB (B) series; Figure S5: PAGE analysis of STAT (A) and STATB (B) series; Figure S6: Kinetics of G4 formation for STATB and its analogues; Figure S7: CD spectra at 37°C of STAT analogues in 10% FBS, registered at different times; Figure S8: CD spectra at 37°C of STATB analogues in 10% FBS, registered at different times. Table S1: Percentages of intact oligo persistent at 37 °C in each sample solution (10% FBS in DMEM) at different time.
