# Supplementary material for: Effects of 5-Methyl-2′-Deoxycytidine in G-Quadruplex Forming Aptamers d(G3C)4 and d[GCG2(CG3)3C]: Investigating the Key Role of the Loops
Source: Biomolecules. 2025 May 23;15(6):753. doi: 10.3390/biom15060753 (PMC12190944; doi:10.3390/biom15060753)
Supplement: Supplementary file 1 [file biomolecules-15-00753-s001.zip › biomolecules-3577556-supplementary.docx]

**Supplementary Material**

**Effects of 5-methyl-2'-deoxycytidine in G-quadruplex forming aptamers d(G_3_C)_4_ and d[GCG_2_(CG_3_)_3_C]: investigating the key role of the loops**

Veronica Esposito, Daniela Benigno, Carla Aliberti, Camilla Esposito, Elisabetta Panza, Antonella Virgilio* and Aldo Galeone.

*Department of Pharmacy, University of Naples Federico II, Via D. Montesano 49, I-80131 Naples, Italy.*

*Corresponding author: Antonella Virgilio; e-mail address: antonella.virgilio@unina.it

**Table of contents**

1. CD spectra.
2. CD melting profiles at high potassium concentration.
3. CD melting profiles at low potassium concentration.
4. 1H-NMR
5. PAGE
6. Kinetics of G4 formation
7. CD spectra in 10% Fetal Bovine Serum (FBS)
8. Table of intact oligo percentages at different times


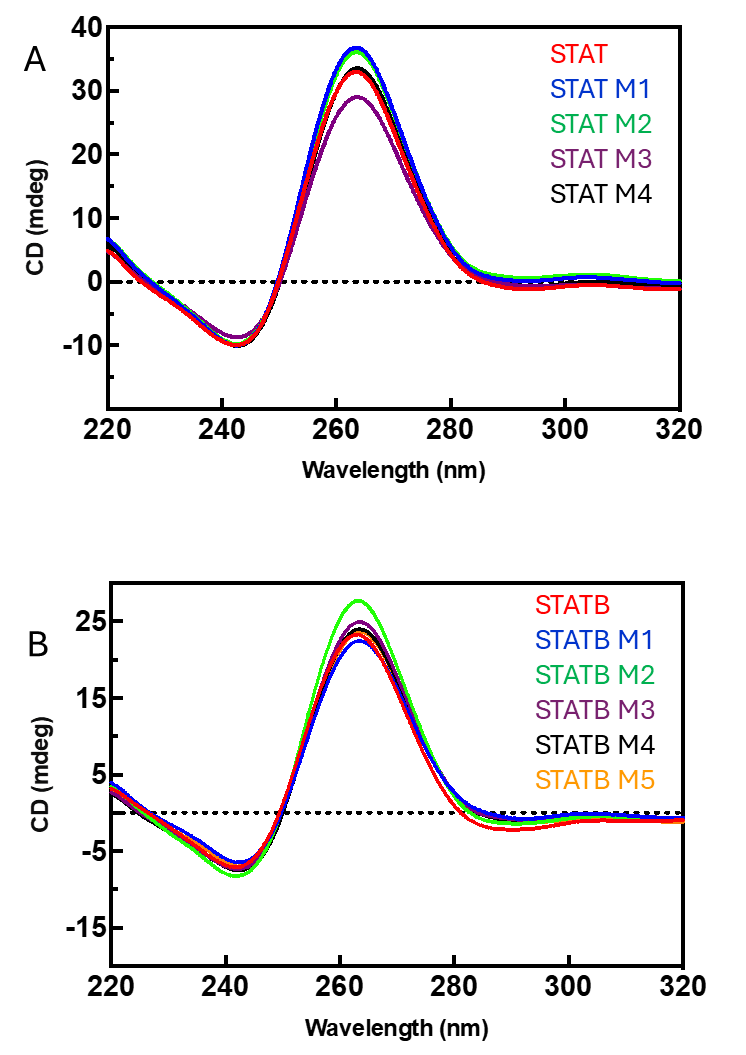


**Figure S1**. CD spectra at 20°C of STAT (A) and STATB (B) analogues in potassium phosphate buffer 2 (1 mM KH_2_PO_4_/K_2_HPO_4_, 5 mM KCl, pH 7.0).


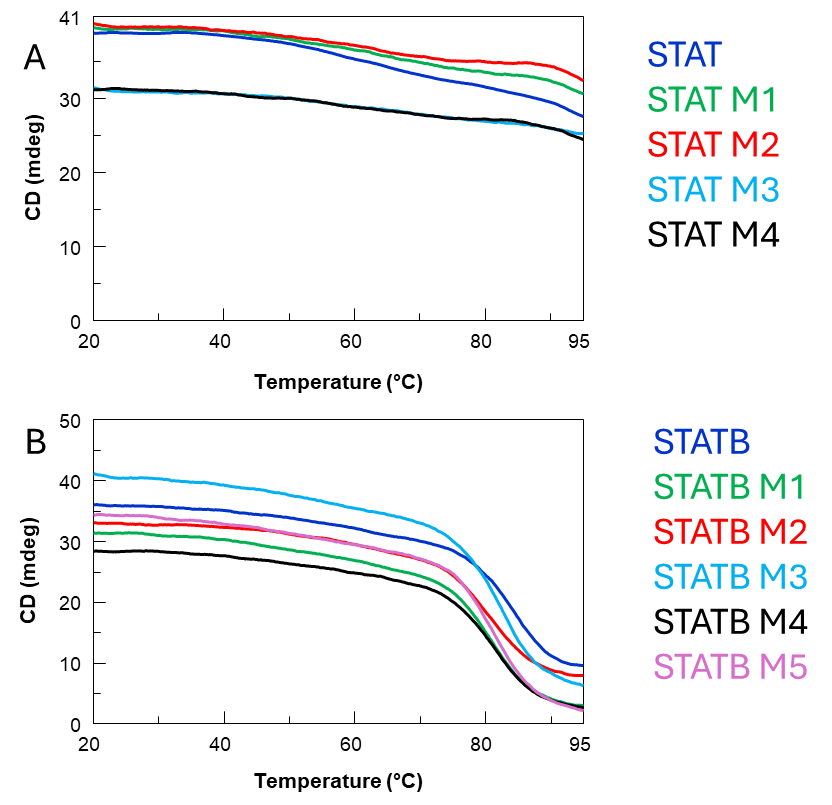


**Figure S2**. CD melting profiles of analyzed ODNs registered as a function of temperature for all modified quadruplexes at their maximum Cotton effect wavelengths. CD data were recorded in a 0.1 cm pathlength cuvette with a scan rate of 30°C/h at 25 µM ODN strand concentration in potassium phosphate buffer 1 (10 mM KH_2_PO_4_/K_2_HPO_4_, 70 mM KCl, pH 7.0).


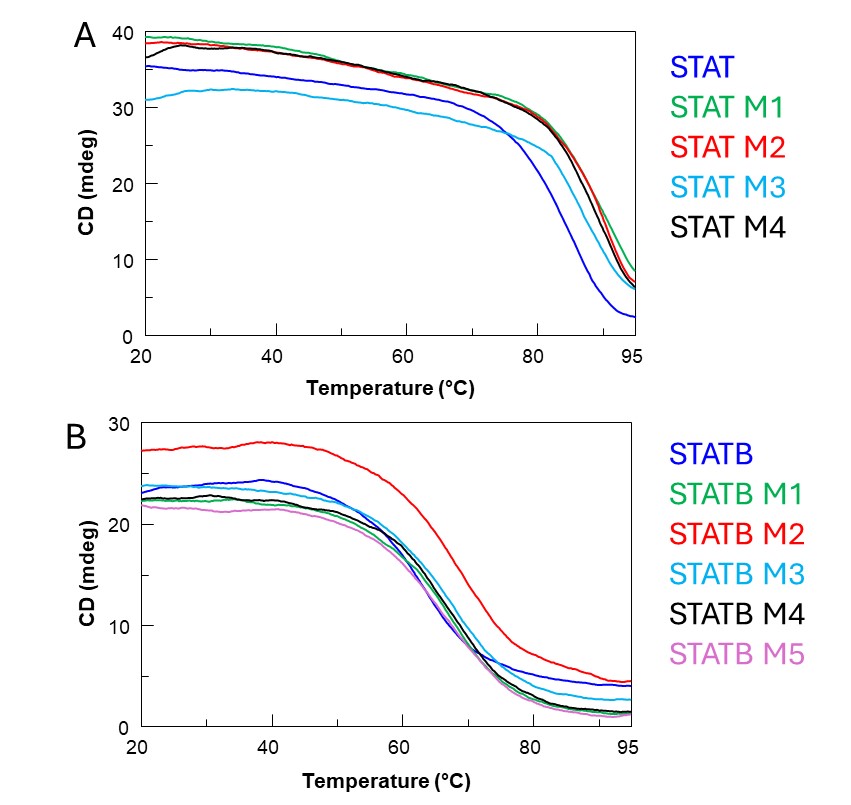


**Figure S3**. CD melting profiles of analyzed ODNs registered as a function of temperature for all modified quadruplexes at their maximum Cotton effect wavelengths. CD data were recorded in a 0.1 cm pathlength cuvette with a scan rate of 30°C/h at 25 µM ODN strand concentration in potassium phosphate buffer 2 (1 mM KH_2_PO_4_/K_2_HPO_4_, 5 mM KCl, pH 7.0).

**
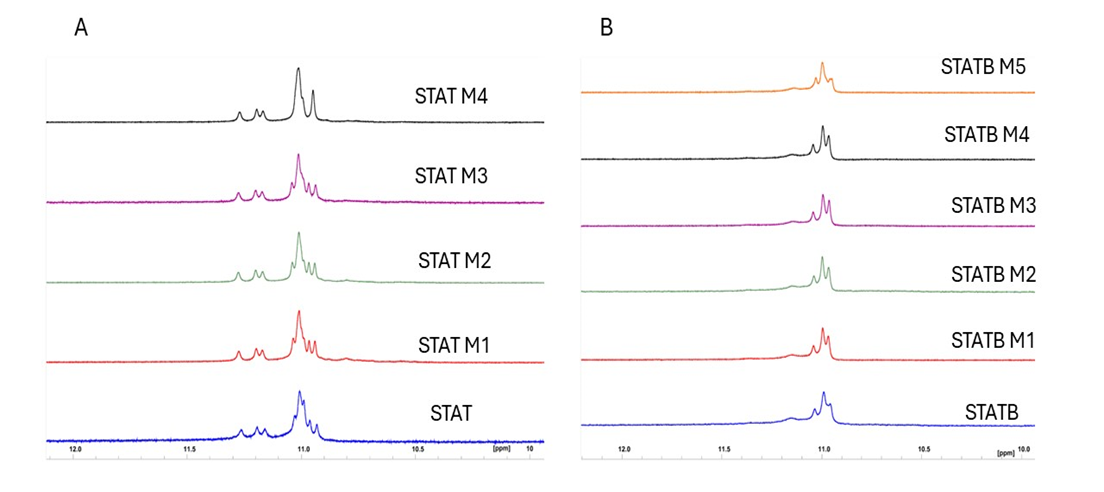
**

**Figure S4.** Imino proton regions of the 1H-NMR spectra (700 MHz) of STAT (A) and STATB (B) series. See Experimental Section for details.


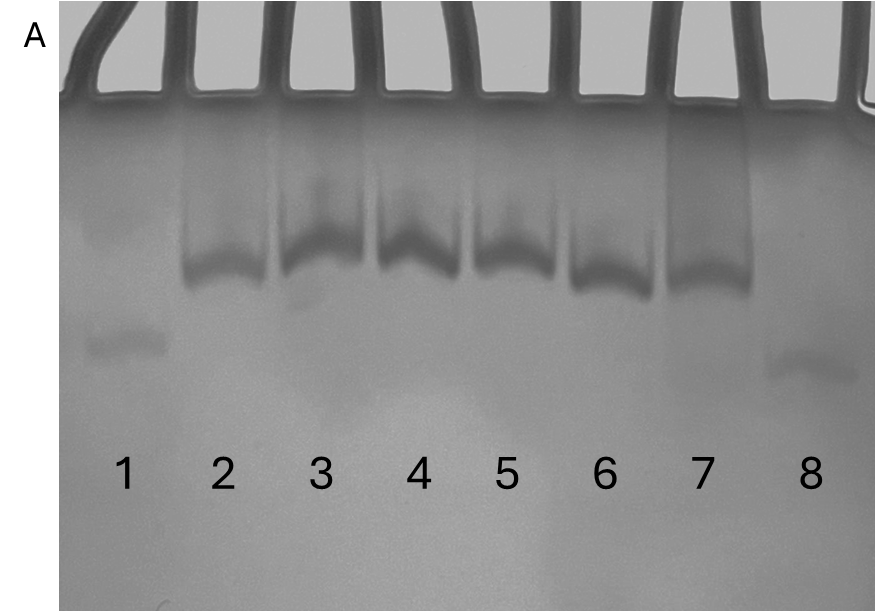


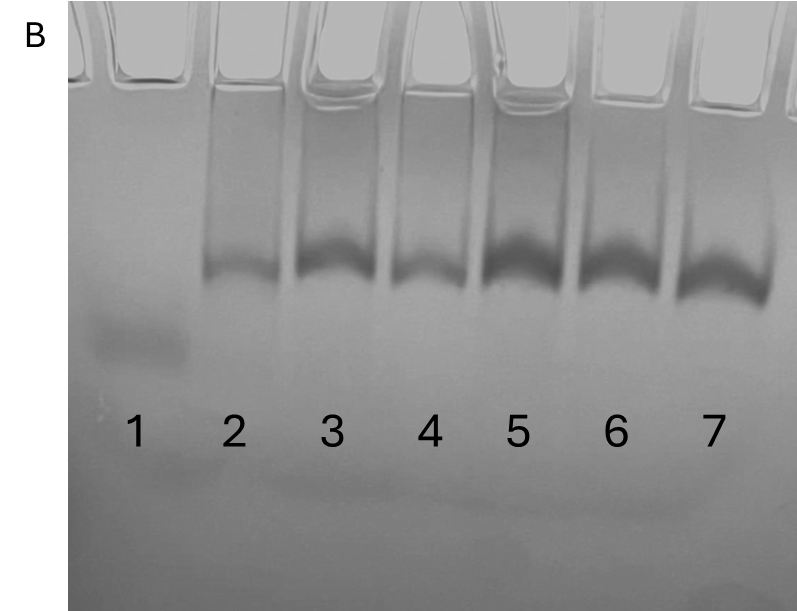


**Figure S5.** PAGE analysis of STAT and its analogues studied (panel A). Lane 1: TTTT- STAT; lane 2: STAT; lane 3: STAT M1; lane 4: STAT M2; lane 5: STAT M3; lane 6: STAT M4; lane 7: STATB; lane 8: TT-STATB. PAGE analysis of STATB and its analogues studied (panel B). Lane 1: TT-STATB; lane 2: STATB; lane 3: STATB M1; lane 4: STATB M2; lane 5: STATB M3; lane 6: STATB M4; lane 7: STATB M5. See the main text and Experimental section for details.


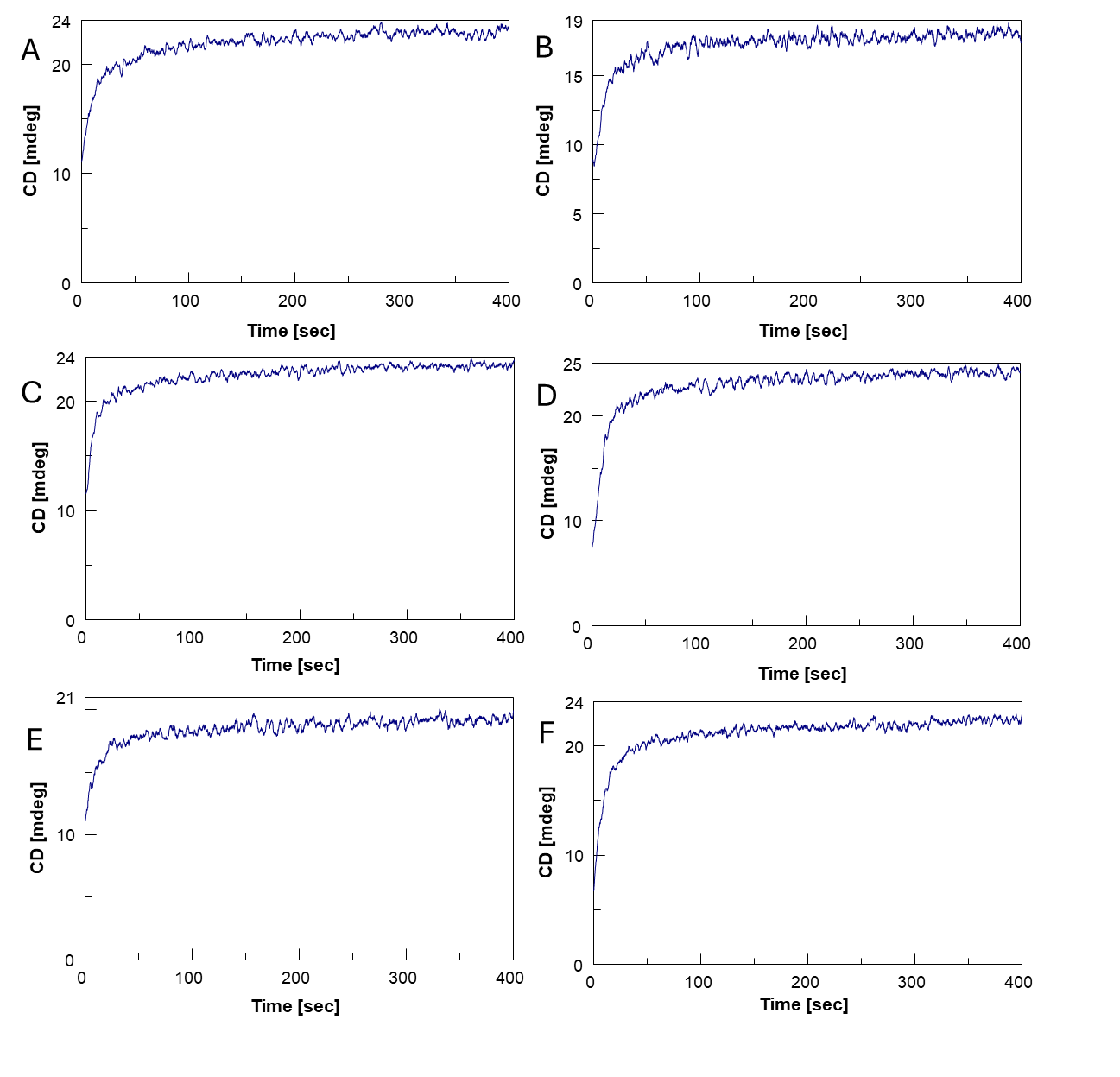


**Figure S6.** Kinetics of G4 formation for STATB (A), STATB M1 (B), STATB M2 (C), STATB M3 (D), STATB M4 (E), STATB M5 (F) at 20 °C in potassium phosphate buffer (1 mM pH 7.0) supplemented with 5 mM KCl (ODN concentration: 25 µM). G4 formation leads to an increase of molar ellipticity at 264 nm.


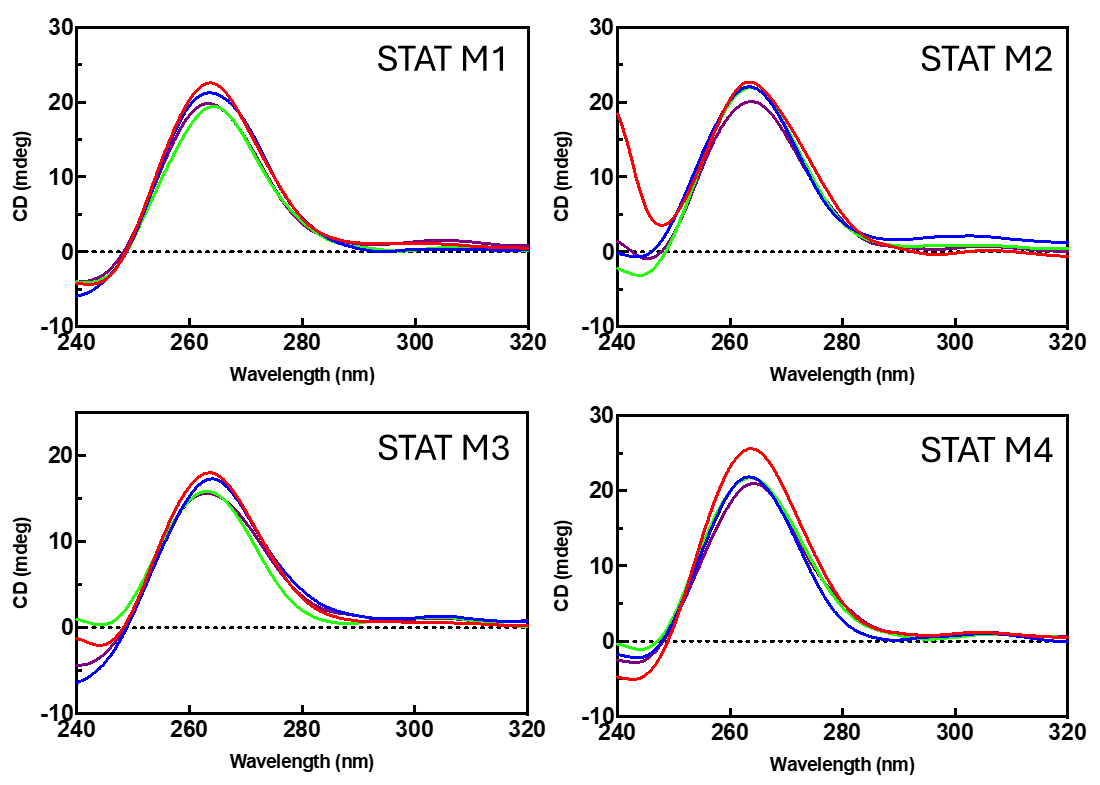


**Figure S7**. CD spectra at 37°C of STAT analogues in 10% Fetal Bovine Serum (FBS) diluted with Dulbecco’s Modified Eagle’s Medium (DMEM), registered at different times: 0 h (red), 24 h (blue), 48 h (green), 72 h (purple). See the main text and the Experimental section for details.


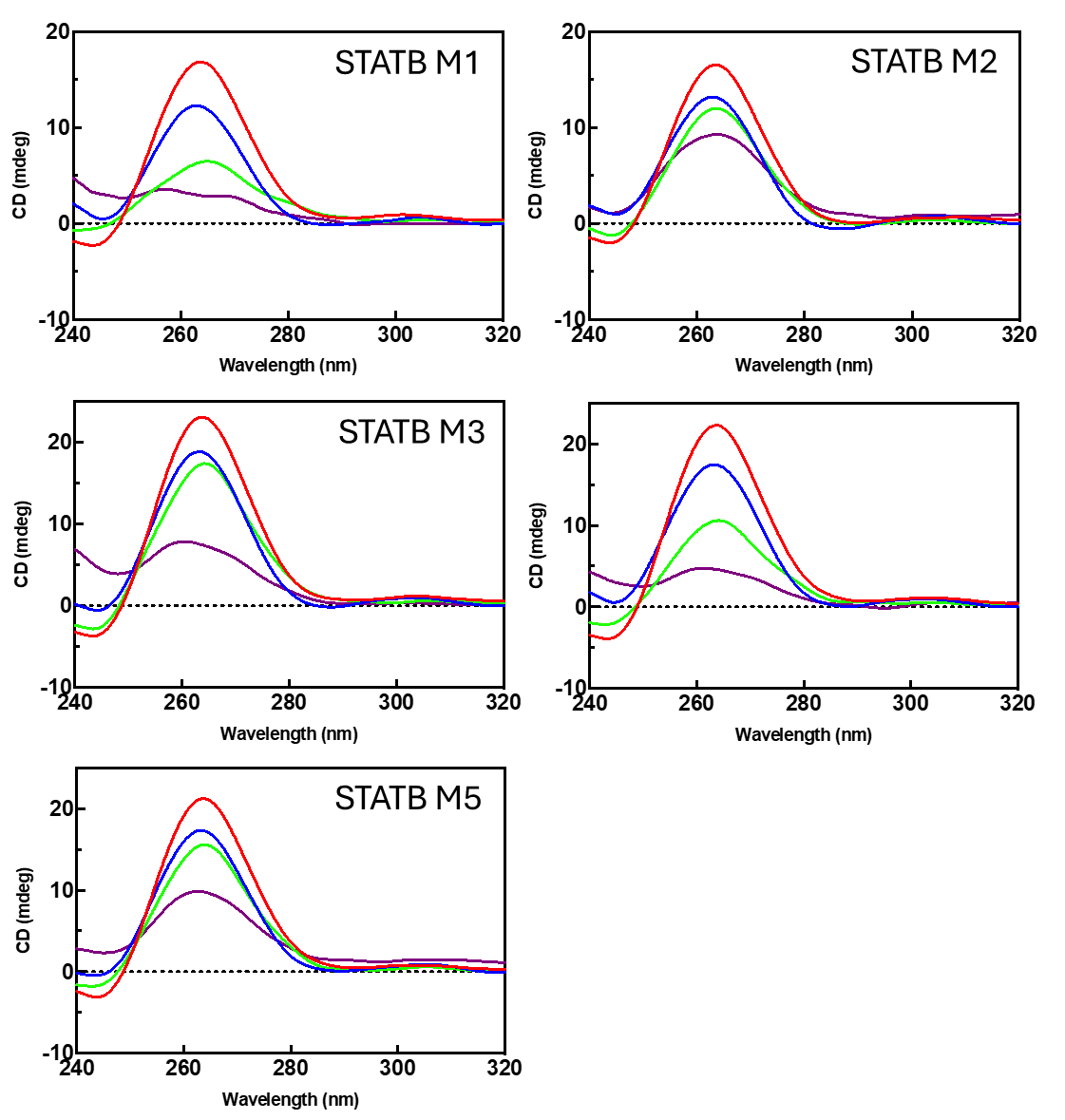


**Figure S8**. CD spectra at 37°C of STATB analogues in 10% Fetal Bovine Serum (FBS) diluted with Dulbecco’s Modified Eagle’s Medium (DMEM), registered at different times: 0 h (red), 24 h (blue), 48 h (green), 72 h (purple). See the main text and the Experimental section for details.


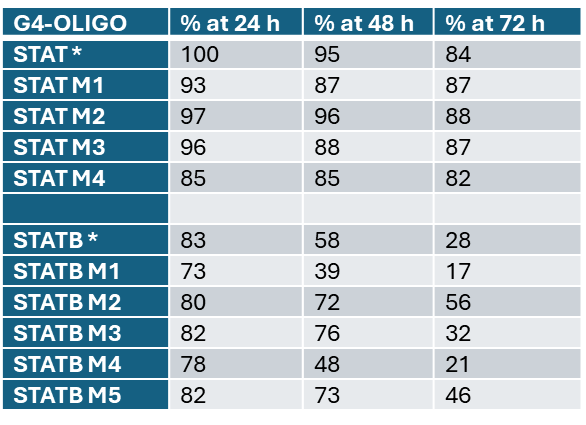


**Table S1.** Percentages of intact oligo persistent at 37°C in each sample solution (10% FBS in DMEM) at different times; *data reported in Int. J. Mol. Sci. 2023, 24, 9524. <https://doi.org/10.3390/ijms24119524>.

All data were repeated for two separate experiments and determined within a reproducibility of 2%.
